# Metformin induces significant reduction of body weight, total cholesterol and LDL levels in the elderly – A meta-analysis

**DOI:** 10.1371/journal.pone.0207947

**Published:** 2018-11-26

**Authors:** Margit Solymár, Ivan Ivic, László Pótó, Péter Hegyi, András Garami, Petra Hartmann, Erika Pétervári, László Czopf, Alizadeh Hussain, Zoltán Gyöngyi, Patrícia Sarlós, Mária Simon, Péter Mátrai, Bálint Bérczi, Márta Balaskó

**Affiliations:** 1 Institute for Translational Medicine, Medical School, University of Pécs, Pécs, Hungary; 2 Institute of Bioanalysis, Medical School, University of Pécs, Pécs, Hungary; 3 Hungarian Academy of Sciences—University of Szeged, Momentum Gastroenterology Multidisciplinary Research Group, Szeged, Hungary; 4 Department of Translational Medicine, First Department of Medicine, Medical School, University of Pécs, Pécs, Hungary; 5 Institute of Surgical Research, University of Szeged, Szeged, Hungary; 6 Department of Cardiology, First Department of Medicine, Medical School, University of Pécs, Pécs, Hungary; 7 Department of Haematology, First Department of Medicine, Medical School, University of Pécs, Pécs, Hungary; 8 Department of Public Health Medicine, Medical School, University of Pécs, Pécs, Hungary; 9 Department of Gastroenterology, First Department of Medicine, Medical School, University of Pécs, Pécs, Hungary; 10 Department of Psychiatry and Psychotherapy, Medical School, University of Pécs, Pécs, Hungary; Medical University of Vienna, AUSTRIA

## Abstract

**Background:**

Metformin is the first-choice drug for patients with Type 2 diabetes, and this therapy is characterized by being weight neutral. However, in the elderly an additional unintentional weight loss could be considered as an adverse effect of the treatment.

**Objectives:**

We aimed to perform a meta-analysis of placebo-controlled studies investigating the body weight changes upon metformin treatment in participants older than 60 years.

**Materials and methods:**

PubMed, EMBASE and the Cochrane Library were searched. We included at least 12 week-long studies with placebo control where the mean age of the metformin-treated patients was 60 years or older and the body weight changes of the patients were reported. We registered our protocol on PROSPERO (CRD42017055287).

**Results:**

From the 971 articles identified by the search, 6 randomized placebo-controlled studies (RCTs) were included in the meta-analysis (n = 1541 participants). A raw difference of -2.23 kg (95% CI: -2.84 –-1.62 kg) body weight change was detected in the metformin-treated groups as compared with that of the placebo groups (p<0.001). Both total cholesterol (-0.184 mmol/L, p<0.001) and LDL cholesterol levels (-0.182 mmol/L, p<0.001) decreased upon metformin-treatment.

**Conclusions:**

Our meta-analysis of RCTs showed a small reduction of body weight together with slight improvement of the blood lipid profile in patients over 60 years. With regard to the risk of unintentional weight loss, metformin seems to be a safe agent in the population of over 60 years. Our results also suggest that metformin treatment may reduce the risk of major coronary events (-4-5%) and all-cause mortality (-2%) in elderly diabetic populations.

## Introduction

Metformin therapy is the initial treatment for patients with Type 2 diabetes according to the current guidelines of the American Diabetes Association/European Association for the Study of Diabetes and the American Association of Clinical Endocrinologists/American College of Endocrinology [1,2]. Metformin is also recommended as a combination therapy for patients with Type 2 diabetes [2]. These recommendations are based primarily on the glucose-lowering effects, relatively low cost, and generally low level of side effects of metformin [3]. Moreover, in contrast to other antidiabetic treatments, metformin seems to be weight neutral or can even help to decrease body weight by decreasing food intake [4,5]. Mechanisms of metformin treatment include reduced gastrointestinal absorption of carbohydrates, as well as decreased insulin and leptin resistance [6], the reduction of plasma ghrelin [7], and induction of lipolysis and anorexia by activation of glucagon–like peptide 1 (GLP-1) [8]. Metformin also reduces ectopic lipid depots in liver and skeletal muscle through increased fat oxidation and decreased lipid synthesis [9].

Metformin seems to be a promising drug for aging prevention in humans [10], since in addition to its anti-diabetic actions, it also exerts anti-tumor and anti-aging effects [11]. Hence, in the future, an increasing number of elderly individuals is expected to take metformin. Therefore, the detailed assessment of the side effects of metformin in the elderly is of extremely high importance.

Currently, about 33% of the population over 65 years have diabetes in the United States [12], and this number is expected to grow rapidly within decades. As the prevalence of Type 2 diabetes is increasing in the elderly, a pressing question of safety arises, as weight loss of the elderly could exaggerate the severity of aging anorexia. This age-related reduction in energy intake leads to severe consequences such as decreased muscle mass and strength (sarcopenia) leading to falls and frailty [13], immobilization, diminished bone density, increased risk of hip fracture [14], longer hospital stay and increased mortality [15]. Because of these potentially severe effects of age-related cachexia, involuntary weight loss—as a possible side effect of metformin—would make this drug unsafe for the elderly population. Indeed, a recent observational study published by the Journal of American Geriatric Society suggested that the use of metformin could lead to severe involuntary weight loss in elderly patients [16].

In 2008, a systematic review and meta-analysis by Golay [5] investigated the effects of metformin on the body weight. That study found no significant weight-reducing effect of metformin compared to placebo either in diabetic or in non-diabetic patients. However, that comprehensive meta-analysis failed to analyze the data of elderly participants separately. It may have been due to a lack of data, because elderly patients are generally underrepresented in clinical trials, although, they are characterized by polypharmacy [17].

Based on the observational study of Pérez-Hernandez and coworkers [16] and on the known mechanisms of action of metformin we hypothesized that metformin treatment reduces body weight in the elderly. The aim of our meta-analysis was to test this hypothesis. Therefore, we conducted a meta-analysis to evaluate the effect of metformin treatment on involuntary weight loss in patients aged 60 or above. This age cutoff for defining elderly or older persons was chosen based on the World Health Organization (WHO) recommendation [18]. We also aimed to analyze other outcome parameters (e.g., Hemoglobin A1c [HbA1c], fasting glucose, total, low-density lipoprotein [LDL], or high-density lipoprotein [HDL] cholesterol, and triglyceride levels or blood pressure values) reported by the articles identified by our search and included in our meta-analysis.

## Materials and methods

### Search strategy for identification of studies

Our meta-analysis was conducted in accordance with the guidelines of the Preferred Reporting Items for Systematic Reviews and Meta-Analysis (PRISMA) Protocols [19]. We registered our protocol with the Prospero Center for Reviews and Dissemination (CRD42017055287).

Meta-analysis was performed using the PICO format: whether administration of metformin (I) compared with placebo (C) has any effect on body weight (primary outcome) and fasting glucose levels, HbA1c, total cholesterol, LDL, HDL, triglyceride levels, systolic and diastolic blood pressure values (secondary outcomes) (O) in human participants where the mean age of the metformin-treated patients was 60 years or above (P). Clinical trials were identified by searching PubMed, EMBASE and Cochrane Library databases from inception until June 2018. In general, the following search terms were used in all databases: "metformin" and "body weight" and ("aged" or "elderly") and "placebo". Specifically, in PubMed we searched the following terms: ("metformin"[MeSH Terms] OR "metformin"[All Fields]) AND ("body weight"[MeSH Terms] OR ("body"[All Fields] AND "weight"[All Fields]) OR "body weight"[All Fields]) AND (("aged"[MeSH Terms] OR "aged"[All Fields] OR "elderly"[All Fields]) OR ("aging"[MeSH Terms] OR "aging"[All Fields]) OR ("aging"[MeSH Terms] OR "aging"[All Fields] OR "ageing"[All Fields])) AND "humans"[MeSH Terms] AND "placebo"[All Fields]. In EMBASE we searched for: ('metformin'/exp OR metformin) AND ('body weight'/exp OR 'body weight') AND ('aged'/exp OR 'aged' OR 'elderly'/exp OR 'elderly' OR 'aging'/exp OR 'aging') AND ('placebo'/exp OR 'placebo'). In the Cochrane Library we used the following search terms "metformin" and "body weight" and ("aged" or "elderly” or “aging” or “ageing”) and "placebo". We included human trials without any restriction to language or year of publication. We have carefully reviewed all articles reporting placebo-controlled metformin studies to check the mean age of the metformin-treated participant group. Two reviewers independently extracted data from all the studies (MS and II) fulfilling the inclusion criteria and any disagreement was resolved by detailed discussion and thus reaching a consensus. Exclusion criteria were based on the lack of proper placebo group even in the presence of a metformin group; mean age of the participants of the metformin group below 60 years of age; lack of body weight data. Data extracted from the papers included: study type, randomization or blinding, number of participants, dosage of metformin, age of participants, study duration, body mass index (BMI), weight gain or loss over time, and other outcome parameters reported in the articles. Fasting glucose levels, total cholesterol, LDL, HDL, triglyceride levels, HbA1c, systolic and diastolic blood pressure changes were presented in at least three of the six studies. The authors of the studies and the year of publication were also recorded.

### Statistical analysis

We have used mean values of paired differences (differences between groups in change from baseline to follow-up) with 95% confidence intervals (CI) as effect size data. The differences were calculated by subtracting paired change after metformin from paired change after placebo. Forest plots were used to describe differences. Between-study heterogeneity was tested with a) Q homogeneity test statistic (p values of less than 0.1 were considered as indicators of significant heterogeneity) and b) I^2^ statistics, where I^2^ is the proportion of total variation attributable to between-study variability (an I^2^ value of more than 50% was considered as indicating considerable heterogeneity). If the Q test is significant, it implies that the heterogeneity among effect sizes reported in the observed studies is more diverse than it could be explained only by random error.

Random effect models were applied in each of the meta-analyses (due to conceptual reasons, even when heterogeneity was small), which were calculated with the DerSimonian and Laird between study variance estimation method [20].

With regard to the presence of small study effect, due to the low number of the included studies, the results of the Egger’s test are not informative; therefore they were omitted from the results.

All analyses were performed with the Comprehensive Metaanalysis software (Biostat, Inc., Engelwood, MJ, USA).

### Quality assessment

The quality of each study was assessed using the Cochrane ‘Risk of bias’ tool [21]. The considered factors included random sequence generation and allocation concealment, description of drop-outs and withdrawals, blinding (participants, personnel, and outcome assessment), the integrity of the results, selective outcome reporting, and other bias [22]. Two independent reviewers (MS and II) assessed the quality of the included studies.

## Results

### Search results

**Fig 1:**
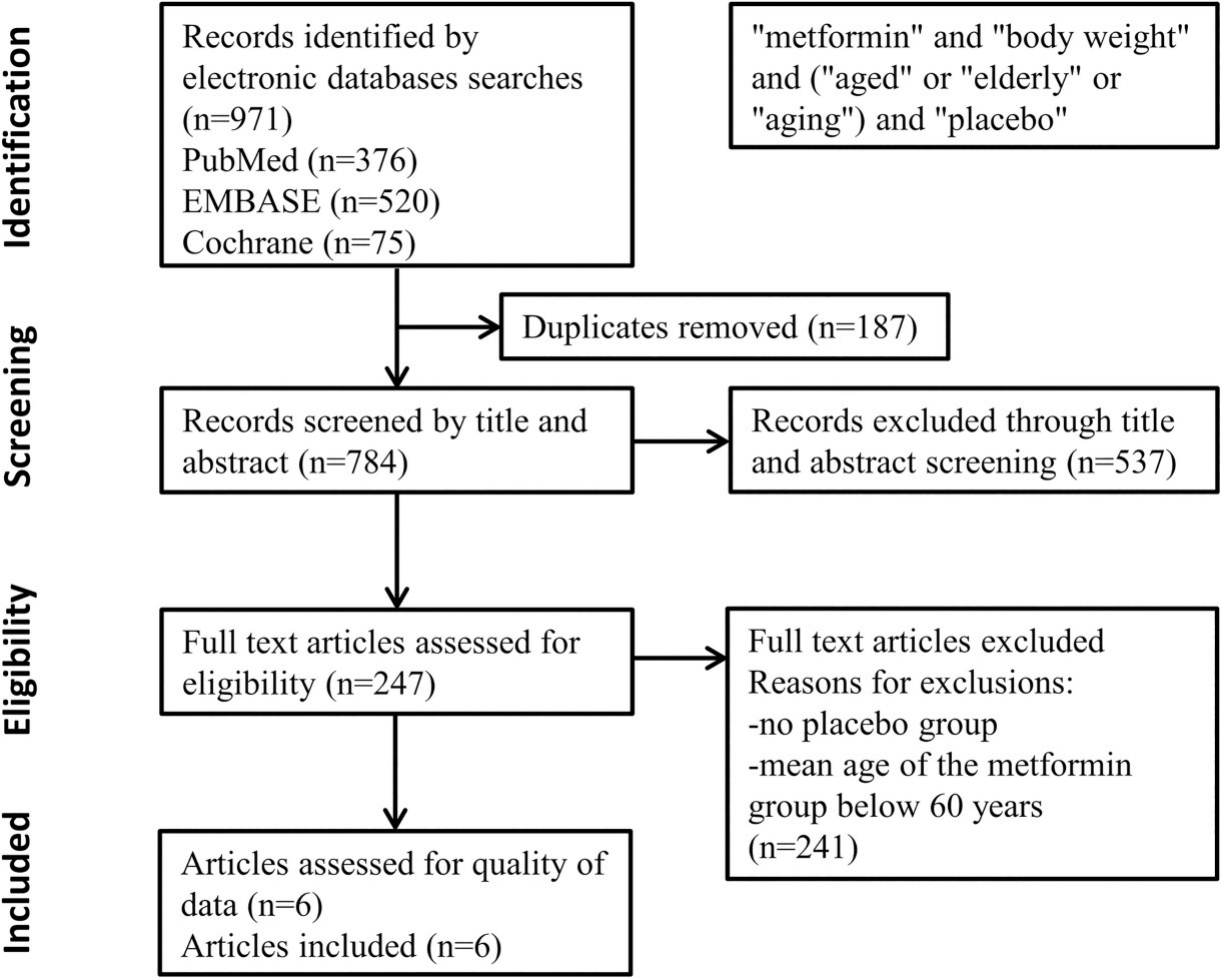
PRISMA flow diagram detailing process of study selection for the meta-analysis.

A total of 971 articles were identified from the database searches. With regard to duplicates, 187 items were removed. Based on title and abstract screening 537 articles were excluded. In case of the remaining 247 articles, full contents were reviewed. Studies were removed where the mean age of the patients with metformin treatment was under 60 years. At the end of the detailed screening process, a total of 6 eligible studies (with 1541 participants) were included in the meta-analysis (**Fig 1**).

### Study characteristics

Six studies were included in the meta-analysis [23–28]. Characteristics of the included studies are summarized in **Table 1**. All studies were randomized controlled trials (RCTs) with placebo control. In five out of the six studies, participants had previously diagnosed Type 2 diabetes, who already received treatment (mostly insulin). These patients received insulin or some oral antidiabetic drugs plus placebo or the corresponding treatment plus metformin. In one study all patients had impaired glucose tolerance and received either placebo or metformin treatment [23]. There was no difference between the initial mean body weight of the patients of the metformin-treated and that of the placebo-treated groups in any of the studies.

**Table 1:** Summary characteristics of the studies included in the analysis.

Study	Study length	Study protocol	Age, mean±SD or age range	Baseline body mass index, mean+SD or interquartile range, IQR	Sample size (metformin/placebo)	Attrition
DPP Research Group, 2006	~ 3.2 years	placebo or metformin 850 mg 2* daily for IGT patients in Diabetes Prevention Program	66.4 (60–85)	30.3 (5.4, IQR) (metformin) 30.8 (7.6, IQR) (placebo)	214/201	ITT, adherent to medication: 71 and 81% (metformin and placebo)
Hermann et al, 1994	6 months	glyburide+placebo vs. glyburide+metformin (500–1500 mg metformin) for patients with Type 2 diabetes	60 (34–74)	no data	46/19	ITT, completers: 89%
Kooy et al, 2009	4.3 years	placebo or metformin 2163/2050 mg 2* daily for insulin-treated patients with Type 2 diabetes	63.6±9.6 (metformin) 59.1±11 (placebo)	30±5 (metformin) 30±5 (placebo)	131/146	ITT, completers: 72%
Lundby-Christensen et al, 2016	18 months	placebo or metformin (2*1 g) to patients with insulin-treated Type 2 diabetes	61±8.7 (metformin) 60.3±9.1 (placebo)	32.3±4.2 (metformin) 32.1±4.2 (placebo)	206/206	ITT, completers: 85 and 76% (metformin and placebo)
Robinson et al, 1998	12 weeks, crossover	placebo or metformin (2*1 g) to patients with poor glycemic control insulin-treated Type 2 diabetes	61.3±7.1	29.5±3.5	19/19	per protocol
Wulffelé et al, 2002	4 months	placebo or metformin (850 mg*2.5 pills) for insulin-treated patients with Type 2 diabetes	63.2±9.8 (metformin) 58.9±11.1 (placebo)	29.9±5.2 (metformin) 29.5±4.6 (placebo)	171/182	per protocol

SD: Standard Deviation, ITT: intention to treat

The durations of the studies were different. Three studies were short term from 12 weeks to 6 months [24,27,28] and three long-term multi-center studies lasted from 18 months to several years [23,25,26]. Significant differences between the mean age of the placebo and metformin groups before randomization were indicated in two of the articles. Patients randomized to metformin treatment were slightly older than patients randomized to placebo in both studies [25,28].

### Risk of bias assessment

**Fig 2:**
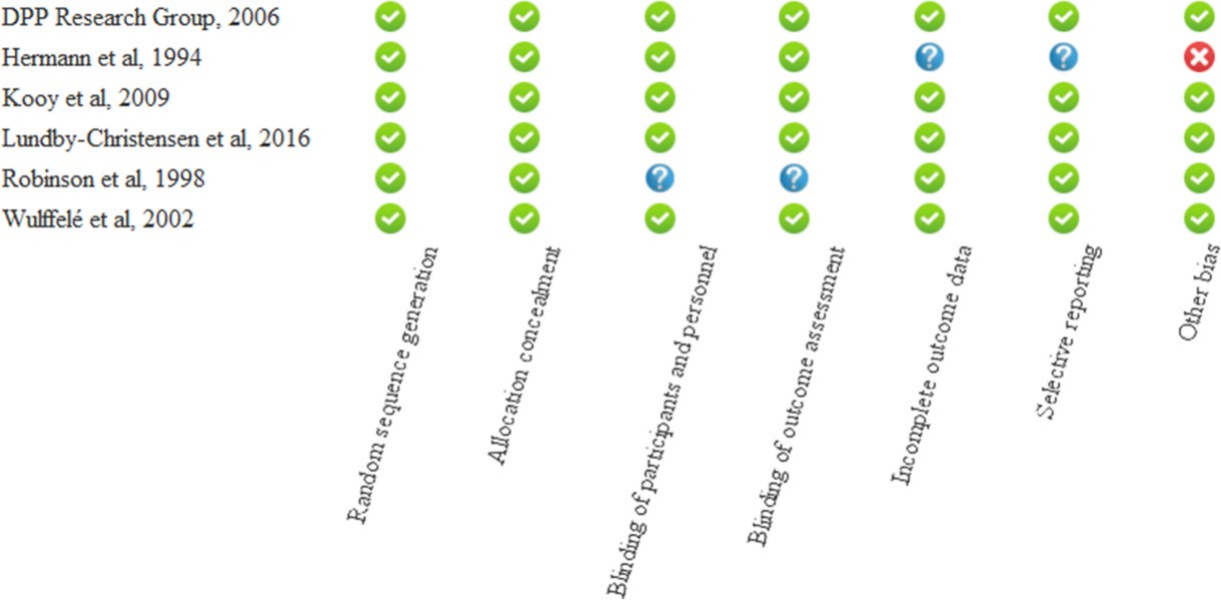
Summary assessment of risk of bias of the included studies.

The details of the risk of bias assessment are summarized in **Fig 2**. Four of the studies received maximal score, and no study was considered low quality. In the study of Hermann et al [24], six different doses of metformin and glyburide were used, therefore outcome data and outcome recordings were not unequivocal, since they reported the pooled results of three dose combinations. Other bias is represented in the same study due to the fact, that the doses of metformin and glyburide were adjusted according to the needs of the patients. In the study of Robinson and coworkers [27], details about blinding were not reported. No subgroup analysis was needed based on study quality. Three studies applied the “Last observation carried forward” method [23, 24, 28], one study imputed missing data by application of the multiple imputation method [26], one study used the summary mean of nonmissing values over the entire observation period [25], whereas Robinson et al provided no information on attrition.

### Effect of metformin treatment on body weight

**Fig 3:**
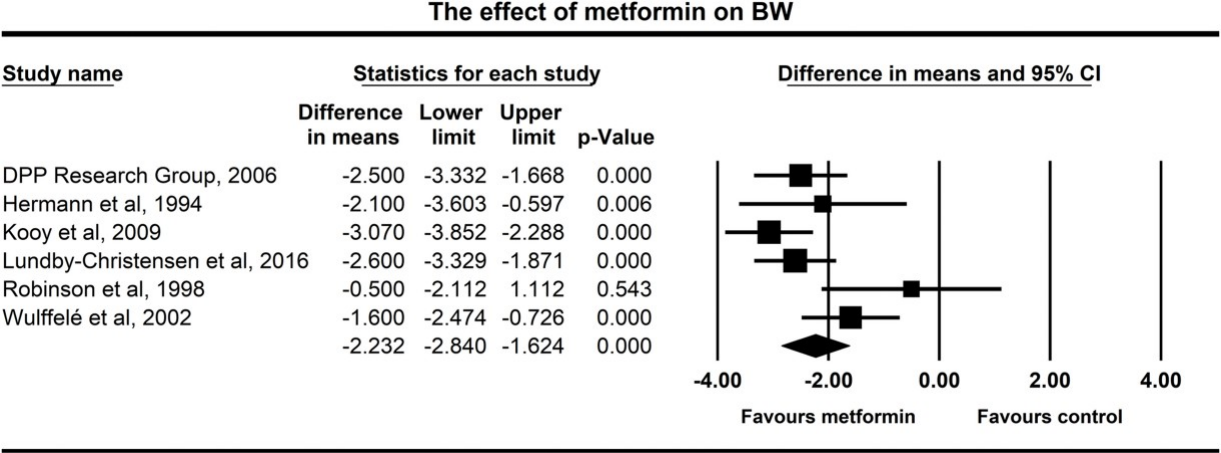
Forest plot analysis of body weight (BW) in participants treated with metformin compared with placebo.

**Fig 4:**
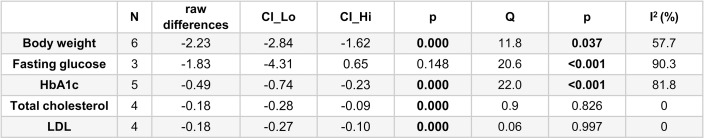
Summary data of outcome parameters.

All of the six studies showed a decrease in body weight in the metformin treated groups. Our meta-analysis revealed a raw difference of -2.23 kg body weight change (95% CI: -2.84 –-1.62 kg) between the metformin-treated and the various placebo groups (p<0.001) (**Fig 3**). The forest plot clearly shows that the length of the intervention increases the body weight change. The longest study conducted by Kooy and coworkers [25] showed the highest difference in the body weight change between the metformin and the placebo groups. In this study, there was a difference of three kilograms between the body weight change upon metformin administration compared to the body weight change of the placebo group after 4.3 years, metformin prevented body weight gain. The study of the Diabetes Prevention Program Research group was the second longest with an average intervention period of 3.2 years [23], where we assessed a 2.5 kg weight loss in the metformin group compared to the placebo group. The study of Lundby-Christensen [26] found a decrease of 2.6 kilograms in the metformin group after 18 months. The shortest study of only 12 weeks reported by Robinson and coworkers [27] revealed just a slight trend of weight loss without a significant difference. According to the Q test, there was significant heterogeneity (p = 0.037) and the I^2^ test value exceeded 50% (57.7%). **Fig 4** shows the data for all significant outcomes.

### Effects of metformin treatment on additionally reported outcome parameters

Fasting glucose values were reported in three studies. The fasting glucose value did not change upon metformin administration (**Fig 4**, p = 0.148). Five out of the six studies contained information about changes of the HbA1c. In the metformin-treated group, HbA1c decreased by an average of 0.49 (95% CI: -0.74 –-0.23) (p<0.001) compared to placebo. We found significant heterogeneity both in the fasting glucose and in the HbA1c analyses (p<0.001).

**Fig 5:**
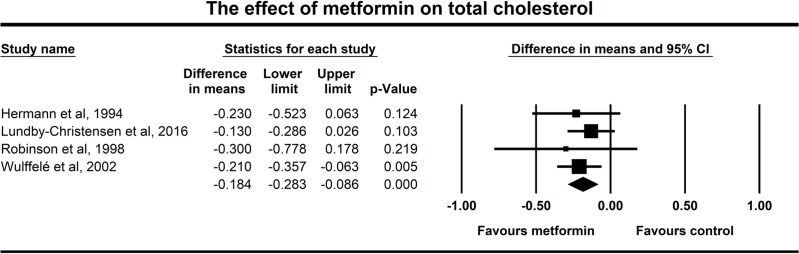
Forest plot analysis of total cholesterol in participants treated with metformin compared with placebo.

Four studies showed changes of total cholesterol upon treatment with metformin. Forest plot for overall effects on total cholesterol level is shown in **Fig 5**. Total cholesterol levels decreased significantly upon the metformin treatment (-0.184 mmol/l, p<0.001). Data of the total cholesterol had particularly low heterogeneity with a non-significant Q test (p = 0.826) and an I^2^ of zero.

**Fig 6:**
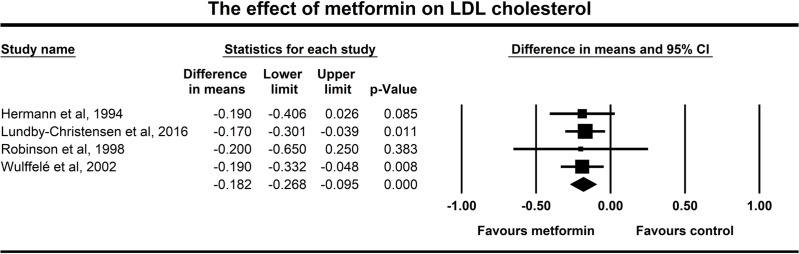
Forest plot analysis of LDL cholesterol in participants treated with metformin compared with placebo.

The same four studies reported also LDL cholesterol changes, the overall effects of metformin on LDL are shown in **Fig 6**. LDL cholesterol declined significantly following the addition of metformin to other treatments (-0.182 mmol/l, p<0.001). Data of the LDL cholesterol had particularly low heterogeneity with a non-significant Q test (p = 0.997) and an I^2^ of zero.

The other outcome parameters reported by the six articles, i.e. changes of HDL (p = 0.648), serum triglycerides (p = 0.349), systolic blood pressure (p = 0.640) or diastolic blood pressure (p = 0.403) did not show any significant change upon metformin administration.

With regard to the adverse effects of metformin, mild gastrointestinal adverse effects/digestive tracts symptoms (e.g. diarrhea, flatulence) appeared more commonly in the metformin group as indicated in four of the six articles [23–25, 28]. With regard to hypoglycemic episodes, in the study of Lundby-Christensen, non-severe hypoglycemia was more common in the metformin group compared to placebo [26]. One study described that discontinuation due to adverse effects was higher in the metformin group (20 metformin, 6 placebo) [28].

## Discussion

In this meta-analysis we found that a long-term metformin intake induces a small, but statistically significant weight loss in participants older than 60. The mean weight loss across studies was slightly above two kilograms, in a population with mean body weights ranging from 80 to 100 (i.e., less than 2.8%) which indicates that metformin treatment may not present a clinically significant risk for unintentional, severe weight loss in the elderly. Our results contradict such previous observations that described a severe weight loss in metformin-treated elderly patients [16]. However, these findings were reported by observational studies [16] or by such RCTs in which weight changes during metformin therapy were compared not to placebo, but to treatment with another anti-diabetic drug [29,30]. In our meta-analysis, all included trials except for DPP study [23] used a placebo group that received the same antidiabetic treatment (e.g. insulin or glyburide) as the metformin group. Thus, the effect of metformin could be assessed clearly on body weight. Moreover, to our knowledge, ours is the first meta-analysis, in which elderly patients were in the primary focus. In the elderly, there is a well-known, dangerous reduction of energy intake with a very complex background, which leads to severe consequences, for example malnutrition with consequent decrease in active tissues leading to sarcopenia, falls [13], frailty, decreased bone density, increased risk of hip fracture [14], longer hospital stay and increased mortality [15].

As secondary outcomes, we have also analyzed the effects of metformin on blood lipid levels from the included studies. Our analysis revealed a small but significant decrease of total blood cholesterol and LDL cholesterol in the metformin treated elderly groups as compared with changes in the placebo groups. No difference was detected in blood triglyceride levels or HDL, thus the decrease of total cholesterol in our meta-analysis is assumed to be due to the decrease in LDL cholesterol. In clinical practice, LDL cholesterol has replaced total cholesterol as a risk marker and the primary treatment target for hyperlipidemias [31]. Reduction in LDL cholesterol has been demonstrated to reduce cardiovascular risk and mortality in a continuous and graded manner over a wide range of LDL cholesterol levels. [32]. The risk of an acute cardiovascular event was elevated by approximately 40% for every 1.0 mmol/L incremental increase in LDL cholesterol [33]. Decreases in the rate of individual endpoints per 1.0 mmol/L LDL cholesterol reductions were major coronary events (24%), coronary revascularization (24%), ischemic stroke (20%), and any stroke (15%) [31]. All-cause mortality was reduced by 10% per 1.0 mmol/L LDL reduction [31]. Based on these epidemiologic findings, our results suggest the metformin treatment may reduce the risk of major coronary events by an estimated 4–5%, and all-cause mortality by about 2% in elderly populations with some abnormality in carbohydrate metabolism. These beneficial changes of the blood lipid profile may be explained by a number of mechanisms [9] some of which can also contribute to the well-known weight reducing effect of metformin. Metformin reduces ectopic lipid depots (i.e. liver and skeletal muscle) through increased fat oxidation and decreased lipid synthesis (for review, see Malin et al, 2014). Wo and coworkers attributed these beneficial effects of metformin in the liver to increased AMP-activated protein kinase activity [34] that is a major cellular regulator of lipid and glucose metabolism. In addition, central and peripheral regulatory mechanisms have also been suggested [9]. Metformin crosses the blood brain barrier and it was demonstrated to act in the hypothalamus to reduce appetite [35]. It suppresses various orexigenic mediators such as neuropeptide Y or agouti-related peptide and induces anorexigenic agents e.g. proopiomelanocortin. Moreover, metformin exerts additional peripheral regulatory mechanisms that improve leptin [36] and insulin sensitivity [37] and increase GLP-1 levels [38,39].

Although elderly individuals have been observed to show significant weight loss due to metformin intake [16], according to our data analysis from placebo-controlled randomized clinical trials, metformin does not cause severe weight reduction in elderly populations (60+). Thus, metformin appears to be a safe agent in such populations. With regard to older populations (e.g. in the oldest old, i.e. 80+), randomized double-blind placebo-control studies focusing on such patient groups would be essential to assess the possible risk of unintentional weight loss. These studies should also determine the percentage of participants that show an at least 10% weight loss during metformin treatment.

### Strengths and limitations

This meta-analysis protocol was registered, and it was reported in accordance with the PRISMA guidelines. Eligibility was determined by the evaluation of two independent reviewers. The risk of bias was assessed by the Cochrane Collaborations’s Tool. Only high-quality placebo-controlled randomized clinical trials were included in our meta-analysis.

With regard to limitations, no subgroup analysis or meta-regression analyses could be performed due to the limited number of available studies. A subgroup analysis based on different age groups could have been informative; however, the mean age of the participants in the metformin groups was just above 60. Additional age groups over 65 and 80 could have considerably increased the value of this meta-analysis. The observed slight difference between the mean age of the placebo and metformin groups before randomization reported in two articles means another limitation. In addition, even well-designed studies such as the DPP failed to stratify their randomization based on age, thus adding to the limitations concerning the influence of age on metformin effects.

The analyzed studies applied various approaches to attrition; nevertheless, the problem how to handle dropout participants presents a considerable limitation also to our meta-analysis. The use of the “Last observation carried forward method” may have influenced our results.

Another source of limitation is derived from the fact, that our search did not include regulatory websites (e.g. that of the Food and Drug Administration) for unpublished data.

As the selected RCTs were performed in Western European countries (Netherland, UK, Denmark and Sweden) or in the United States (Diabetes Prevention Program), the results of the current study are somewhat limited in generalizability. In the future, similar investigations should be encouraged in Asian, African, Southern-American regions, as well.

Originally, we intended to study the effects of metformin on body weight independently of the underlying disease or the reason why metformin was prescribed (e.g. in polycystic ovary syndrome or prostate cancer), however due to the lack of placebo-controlled trials focusing on such diseases, all patients that we could include suffered of Type 2 diabetes or impaired glucose tolerance.

With regard to the secondary analysis of other than body weight, i.e. lipid parameters, fasting glucose values, HbA1c or blood pressure values, none of our original search terms focused on them. As our main goal was to assess the body weight changes, we included only those studies in which body weight changes were reported. However, based on our current results, a new analysis would be necessary for further assessment of changes of blood lipid parameters in metformin treatment.

## Conclusions

Metformin intake in the elderly is associated with improved glycemic control, and moderate diminishment of weight gain or a small weight loss together with significant decrease in total serum cholesterol and in serum LDL cholesterol. Our analysis confirmed that metformin is a safe agent for the treatment of Type 2 diabetes in the 60+ age group. In addition, based on the observed reduction in LDL cholesterol our study suggests that metformin treatment may reduce the risk of major coronary events and all-cause mortality in elderly diabetic populations.

## Supporting information

S1 File PRISMA 2009 checklist. PRISMA: Preferred Reporting Items for Systematic Reviews and Meta-Analyses.
(PDF)

S2 File Protocol of this meta-analysis as it is registered on PROSPERO. (PDF)

S3 File Original raw data reported by the selected articles. (PDF)
