# Supplementary material for: Metformin induces significant reduction of body weight, total cholesterol and LDL levels in the elderly – A meta-analysis
Source: PLoS One. 2018 Nov 26;13(11):e0207947. doi: 10.1371/journal.pone.0207947 (PMC6258123; doi:10.1371/journal.pone.0207947)
Supplement: S2 File — (PDF) [file pone.0207947.s002.pdf]

Meta-analysis of body weight changes after metformin intake in the elderly

Margit Solymár, Ivan Ivic, László Pótó, Péter Hegyi, András Garami, Imre Szabó, Petra Hartmann, Erika Pétervári, Márta Balaskó, Gábor Veres, László Czakó, Judit Bajor, László Czopf, Péter Mátrai

Citation

Margit Solymár, Ivan Ivic, László Pótó, Péter Hegyi, András Garami, Imre Szabó, Petra Hartmann, Erika Pétervári, Márta Balaskó, Gábor Veres, László Czakó, Judit Bajor, László Czopf, Péter Mátrai. Meta-analysis of body weight changes after metformin intake in the elderly. PROSPERO 2017 CRD42017055287 Available from:
http://www.crd.york.ac.uk/PROSPERO/display_record.php?ID=CRD42017055287

Review question

Does metformin induce weight loss in the elderly?
How does metformin intake influence body mass index after the age of 60 years?

Searches

Literature searches are conducted through EMBASE, PubMed, and Cochrane Library databases. Human studies published in English will be included.

Types of study to be included

At the time of registration there are no restrictions.

Condition or domain being studied

Body mass index and body weight changes.

Participants/population

Elderly people (older than 60 years old) taking metformin for at least 12 weeks.

Intervention(s), exposure(s)

We include data from studies concerning the effects of an at least 12 weeks long metformin intake on the body weight or BMI levels of elderly (>60 years of age) people.

Comparator(s)/control

Placebo-control.

Context

Primary outcome(s)

Change in body weight or body mass index.

Secondary outcome(s)

None.

Data extraction (selection and coding)

Risk of bias (quality) assessment

To assess the presence of publication bias, we use Egger's test to detect asymmetry in the funnel plot. A significant test result ($p < 0.1$) indicates the existence of bias.

Strategy for data synthesis

Forest plots will be used for presenting results together with a narrative synthesis of the studies. If not provided, data will be calculated or estimated where appropriate. Heterogeneity will be calculated using the I-

squared statistic.

Analysis of subgroups or subsets

If the necessary data are available, subgroup analyses will be done.

Contact details for further information

Margit Solymár MD PhD
margit.solymar@aok.pte.hu

Organisational affiliation of the review

University of Pécs, Medical School, Hungary

Review team members and their organisational affiliations

Dr Margit Solymár. Institute for Translational Medicine, University of Pécs, Pécs, Hungary
Mr Ivan Ivic. Institute for Translational Medicine, University of Pécs, Pécs, Hungary
Dr László Pótó. Institute of Bioanalysis, University of Pécs, Pécs Hungary
Professor Péter Hegyi. Institute for Translational Medicine, University of Pécs, Pécs, Hungary
Dr András Garami. Institute for Translational Medicine, University of Pécs, Pécs, Hungary
Dr Imre Szabó. Department of Gastroenterology, First Department of Medicine, University of Pécs, Pécs, Hungary
Dr Petra Hartmann. Institute of Surgical Research, University of Szeged, Szeged, Hungary
Dr Erika Pétervári. Institute for Translational Medicine, University of Pécs, Pécs, Hungary
Dr Márta Balaskó. Institute for Translational Medicine, University of Pécs, Pécs, Hungary
Dr Gábor Veres. 1st Department of Paediatrics, Semmelweis University, Budapest Hungary
Dr László Czakó. First department of Medicine, University of Szeged, Szeged, Hungary
Dr Judit Bajor. Department of Gastroenterology, First Department of Medicine, University of Pécs, Pécs, Hungary
Dr László Czopf. Department of Cardiology, First Department of Medicine, University of Pécs, Pécs, Hungary
Mr Péter Mátrai. Institute of Bioanalysis, University of Pécs, Pécs Hungary

Anticipated or actual start date

15 November 2016

Anticipated completion date

31 August 2018

Funding sources/sponsors

Medical School, University of Pécs, Hungary
PTE-AOK-KA-2016/14 and PTE-AOK-KA-2017

Conflicts of interest

None known

Language

English

Country

Hungary

Stage of review

Review_Completed_not_published

Subject index terms status

Subject indexing assigned by CRD

Subject index terms

Aged; Humans; Hypoglycemic Agents; Metformin; Weight Gain

Date of registration in PROSPERO

13 January 2017

Date of publication of this version

18 July 2018

Details of any existing review of the same topic by the same authors

Stage of review at time of this submission

Stage	Started	Completed
Preliminary searches	Yes	Yes
Piloting of the study selection process	Yes	Yes
Formal screening of search results against eligibility criteria	Yes	Yes
Data extraction	Yes	Yes
Risk of bias (quality) assessment	Yes	Yes
Data analysis	Yes	Yes

Versions

13 January 2017
25 January 2017
18 July 2018

PROSPERO
This information has been provided by the named contact for this review. CRD has accepted this information in good faith and registered the review in PROSPERO. CRD bears no responsibility or liability for the content of this registration record, any associated files or external websites.
