# Supplementary material for: Metformin induces significant reduction of body weight, total cholesterol and LDL levels in the elderly – A meta-analysis
Source: PLoS One. 2018 Nov 26;13(11):e0207947. doi: 10.1371/journal.pone.0207947 (PMC6258123; doi:10.1371/journal.pone.0207947)
Supplement: S3 File — (PDF) [file pone.0207947.s003.pdf]

**Study**	DPP Research Group, 2006				Kooy et al., 2009				Hermann et al., 1994				Wulffelé et al., 2002				Robinson et al., 1998			Lundby-Christensen et al., 2016			
**Randomization**	yes				yes				yes				yes				yes			yes			
**Blinding**	NA				double-blind				double-blind				double-blind				double-blind			yes			
**Substance**	metformin 850 mg 2*daily	metformin 850 mg 2*daily	placebo	placebo	baseline before metformin began	after 4,3 yrs	baseline before placebo	after 4,3 yrs placebo	baseline before metformin	after 6 months	before placebo	after	metformin baseline	metformin after 4 mo	placebo baseline	placebo after 4 mo	baseline insulin	change after 12 weeks metformin + insulin	change after 12 weeks placebo + insulin	metformin baseline	metformin	placebo baseline	placebo
**Number of patients**	214		201		196	131	194	146	46	46	19	19	171	171	182	182	19	19	19	206		206	
**Mean BMI kg/m2**					30	30	30	31					29.9	29.7	29.5	30				32.3		32.1	
**BMI +-SD**					5	5	5	5					5.2	5.3	4.6	5.4				4.2		4.2	
**BW change**		-2.7		-0.2						0.7		2.8		-0.4		1.2		-0.5	0		1.6		4.2
**BW change SD**														2.5		5.3		3.1	1.8				
**BW change SEM**		0.3		0.3						0.4		0.7											
**BW change 95% CI lo**																					1.1		3.6
**BW change 95% CI hi**																					2.1		4.7
**Difference in mean between the groups**																			0.5				-2.6

**To the difference CI lo**																			-0.1				-3.3
**To the difference CI hi**																			2.1				-1.8
**Summary mean**						85		90															
**SD**						16		16															
**Mean difference between the two summary mean**							-3.07																
**To the difference CI lo**							-3.85																
**To the difference CI hi**							-2.28																
**BW mean**					85	87	87	91	80.2	81	82.6	86.2	85.6	85.1	86.2	87.4	81.1	80.6	81.1	97.2		97.1	
**BW SD**					16	17	15	17					15.7	16	14.6	16.1	16.9	3.1	1.8	15.2		14.7	
**BW SEM**									2.4	2.5	2.7	3.3											
**HbA1C mean**					7.9	7.70	7.90	7.90	6.8	5.6	6.7	5.3	7.86	6.94	7.88	7.61	8.9	7.8	9.4				
**HbA1C SD**					1.2	1.10	1.20	1.10					1.17	0.98	1.21	1.17	1	1.3	0.9				
**HbA1c SE**									0.1	0.1	0.3	0.1											
**HbA1c change mean**										-1.2		-1.3		-0.91		-0.27		-1.1	0.5		-0.78		-0.36
**SD**														0.93		0.84		1.3	0.9				
**SE**										0.1		0.2											
**HBA1c change CI 95 % low**																					-0.92		-0.5
**HBA1c change CI 95 % high**																					-0.64		-0.22

**Difference mean between the groups**																		1.6				-0.42	
**To the difference CI low**																		0.9				-0.62	
**To the difference CI high**																		2.4				-0.23	
**Summary mean**						7.50		7.90															
**SD**						1.00		1.00															
**Mean difference between the two summary mean**							-0.40																
**To the difference CI low**							-0.55																
**To the difference CI high**							-0.25																
**Fasting glucose (mmol/l) mean**					8.6	8.1	8.8	8	9.3	7.2	8.6	6.6					11.8	8	13.7				
**Fasting glucose (mmol/l) SD**					1.8	1.6	1.8	1.5									3.7	3.2	3.8				
**Fasting glucose (mmol/l) SE**									0.4	0.2	0.4	0.2											
**Fasting glucose (mmol/l) change mean**										-2.1		-2.1						-3.8	1.9		-2.14		-1.67
**SD**																		3.2	3.8				
**SE**										0.4		0.5											

**Fasting glucose change 95% CI low limit**																					-2.64		-2.18
**Fasting glucose change 95% CI high limit**																					-1.64		-1.16
**Difference between the groups**																		5.8				-0.47	
**To the difference CI low**																		3.5				-1.18	
**To the difference CI high**																		8.1				0.25	
**Total cholesterol mmol/l mean**					5.6	4.2	5.5	4.2	5.46	5.36	5.66	5.78	5.58	5.31	5.49	5.45	6	5.7	6				
**Total cholesterol mmol/l SD**					1.3	0.7	1.3	0.8					1.13	0.99	1.23	1.23	1.1	0.7	0.8				
**Total cholesterol mmol/l SE**									0.16	0.13	0.17	0.15											
**Total cholesterol (mmol/l) chage mean**										-0.1		0.13		-0.25		-0.04		-0.3	0		0.07		0.2
**SD**														0.65		0.75		0.7	0.8				
**SE**										0.08		0.13											

**Total chol change 95 CI low**																					-0.04		0.09
**Total chol change 95 CI high**																					0.18		0.32
**Difference between the groups**																		0.3				-0.13	
**To the difference CI low**																		-0.2				-0.29	
**To the difference CI high**																		0.8				0.03	
**LDL mmol/l mean**					3.6	2.1	3.4	2.2	3.67	3.58	3.93	4.04	3.54	3.27	3.4	3.37	3.9	3.7	3.9				
**LDL mmol/l SD**					1.1	0.6	1	0.6					1.03	0.98	1.02	1.14	1.2	0.6	0.8				
**LDL mmol/l SE**									0.14	0.12	0.14	0.13											
**LDL (mmol/l) change mean**										-0.07		0.12		-0.24		-0.05		-0.2	0		0.04		0.21
**SD**														0.63		0.72		0.6	0.8				
**SE**										0.06		0.09											
**LDL change 95% CI low**																					-0.06		0.12
**LDL change 95% CI high**																					0.13		0.31

**Difference between the groups**																		0.3				-0.18	
**To the difference CI low**																		-0.2				-0.31	
**To the difference CI high**																		0.7				-0.04	
**Plasma triglyceride mmol/l mean**					1.7	1.5	1.9	1.6	1.97	1.89	2.01	2.1	1.66	1.63	1.88	1.9	2.2	3.1	3.2				
**Plasma triglyceride mmol/l SD**					1.2	0.9	1.5	1.6					1.13	1.13	1.53	1.53	1.3	0.2	0.4				
**Plasma triglyceride mmol/l SE**									0.23	0.2	0.37	0.35											
**Triglycerides change mean**										0.06		0.08		-0.02		0.02		0.9	1				
**SD**														0.87		1.21		0.2	0.4				
**SE**										0.13		0.13											
**Difference between the groups**																		0.1					
**To the difference CI low**																		-0.2					
**To the difference CI high**																		0.3					
**HDL mmol/l mean**					1.3	1.35	1.25	1.3	0.91	0.95	0.89	0.92	1.31	1.3	1.26	1.26	1.1	1.1	1.2				

**HDL mmol/l SD**					0.4	0.4	0.4	0.4					0.41	0.39	0.41	0.4	0.3	0.1	0.2				
**HDL mmol/l SE**									0.04	0.05	0.05	0.05											
**HDL mmol/l change mean**										0.04		0.03		-0.01		0		0	0.1		0.01		0
**SD**														0.18		0.17		0.1	0.2				
**SE**										0.03		0.02											
**HDL change 95% CI low**																					-0.02		-0.03
**HDL change 95% CI high**																					0.04		0.02
**Difference between the groups**																		0.1				0.02	
**To the difference CI low**																		-0.1				-0.03	
**To the difference CI high**																		0.2				0.06	
**Systolic BP mean**					160	141	159	141	143	144	141	140	160	161	159	161	138	134	140				
**Systolic BP SD**					25	18	25	14					26	25	25	25	16	13	23				
**Systolic BP SE**									3	3	3	3											
**Systolic BP change mean**										1		-1		1.8		2.1		-4	2		-5.7		-5.4
**SD**														17		17		13	23				
**SE**										2.4		2											

**Systolic BP change 95% CI low**																				-8		-7.7
**Systolic BP change 95% CI high**																				-3.4		-3
**Difference between the groups**																	6				-0.3	
**To the difference CI low**																	-11				-3.6	
**To the difference CI high**																	23				2.9	
**Summary mean**					153		154															
**SD**					18		17															
**Mean difference between the two summary mean**						-0.51																
**To the difference CI lo**						-2.76																
**To the difference CI hi**						1.44																
**Diastolic BP mean**				86	77	86	79	83	85	84	84	85	88	86	88	78	75	82				
**Diastolic BP SD**				12	11	11	10					12	13	11	14	9	9	11				
**Diastolic BP SE**								1	1	1	2											
**Diastolic BP change mean**									2.1		0.5		2.5		2.6		-3	4		-3.5		-3
**SD**													11		11		9	11				
**SE**									1.3		1.2											

**Diastolic BP change 95% CI low**																					-4.8		-4.2
**Diastolic BP change 95% CI high**																					-2.3		-1.7
**Difference between the groups**																		7				-0.6	
**To the difference CI low**																		0				-2.3	
**To the difference CI high**																		15				1.2	
**Summary mean**						85		85															
**SD**						9		9															
**Mean difference between the two summary mean**							-0.88																
**To the difference CI lo**							-3.21																
**To the difference CI hi**							1.45																
